# Comparative chest computed tomography findings of non-tuberculous mycobacterial lung diseases and pulmonary tuberculosis in patients with acid fast bacilli smear-positive sputum

**DOI:** 10.1186/1471-2466-14-65

**Published:** 2014-04-22

**Authors:** Mei-Kang Yuan, Cheng-Yu Chang, Ping-Huang Tsai, Yuan-Ming Lee, Jen-Wu Huang, Shih-Chieh Chang

**Affiliations:** 1 Department of Radiology, National Yang-Ming University Hospital, Yilan, Taiwan; 2 Faculty of Medicine, School of Medicine, National Yang-Ming University, Taipei, Taiwan; 3 Department of Chest Medicine, Far Eastern Memorial Hospital, Taipei, Taiwan; 4 Department of Internal Medicine, National Yang-Ming University Hospital, #152, Xin-Min Road, Yilan City 260, Taiwan; 5 Department of Laboratory Medicine, National Yang-Ming University Hospital, Yilan, Taiwan; 6 Department of Surgery, National Yang-Ming University Hospital, Yilan, Taiwan

**Keywords:** Nontuberculous mycobacterial lung disease, Pulmonary tuberculosis, Acid-fast bacilli smear, Bronchiectasis and cystic changes

## Abstract

**Background:**

Early diagnosis and treatment of nontuberculous mycobacterial lung diseases (NTM-LD) and pulmonary tuberculosis (PTB) are important clinical issues. The present study aimed to compare and identify the chest CT characteristics that help to distinguish NTM lung disease from PTB in patients with acid-fast bacilli (AFB) smear-positive sputum.

**Methods:**

From January 2009 to April 2012, we received 467 AFB smear-positive sputum specimens. A total of 95 CT scans obtained from the 159 patients were analyzed, 75 scans were from patients with PTB and 20 scans from NTM-LD. The typical chest CT findings of mycobacterial diseases were analyzed.

**Results:**

In patients with PTB, the prevalence of pleural effusion (38.7% vs. 15.0%; *P* =0.047), nodules < 10 mm in size (76.0% vs. 25.0%; *P* < 0.001), tree-in-bud pattern (81.3% vs. 55.0%; *P* =0.021), and cavities (31.1% vs. 5.0%; *P* =0.018) were significantly higher than patients with NTM. Of the 20 patients with NTM lung diseases, bronchiectasis and cystic changes were significantly higher than patients with PTB (20.0% vs. 4.0%; *P* = 0.034). In multivariate analysis, CT scan findings of nodules was independently associated with patients with diagnoses of PTB (odds ratio [OR], 0.07; 95% confidence interval [CI], 0.02-0.30). Presence of bronchiectasis and cystic changes in CT scans was strongly associated with patients with NTM-LD (OR, 33.04; 95% CI, 3.01-362.55).

**Conclusions:**

The CT distinction between NTM-LD and PTB may help radiologists and physicians to know the most likely diagnoses in AFB-smear positive patients and avoid unnecessary adverse effects and the related costs of anti-TB drugs in endemic areas.

## Background

The diagnosis and treatment of pulmonary diseases caused by mycobacterial infections are very important clinical issues. Among mycobacterial diseases, pulmonary tuberculosis (PTB) is the major entity and one of the world’s leading infectious diseases. In 2012, 8.6 million people felt ill with TB and 1.3 million died from TB [1]. Isolation of Mycobacterium tuberculosis from respiratory specimens is recommended for the definite diagnosis of PTB [2]. Microscopic examination of sputum smears for acid-fast bacilli (AFB) is widely used and the most efficient procedure for the initial screening of PTB. The presence of AFB in the stained sputum (AFB smear-positive) indicates a preliminary diagnosis of pulmonary mycobacterial infection. However, positive AFB smear test is not specific for PTB [3-5]. AFB smear-positive sputum may represent mycobacterium tuberculosis, but it can also represent non-tuberculous mycobacteria (NTM) [6]. NTM are ubiquitous organisms and its radiographic abnormalities and clinical symptoms changes slowly as compared with PTB [7]. In endemic areas, it is not uncommon to administer empirical anti-TB drugs in clinically suspected PTB patients with AFB smear-positive sputum. The isolation prevalence of NTM has increased gradually, which raise the concerns of unnecessary adverse effect and costs of anti-TB drugs [8,9]. Therefore, additional diagnostic tools that help differentiate PTB and NTM is relevant in patients pending culture results. Radiological manifestations such as computed tomography (CT) features are useful to aid the diagnosis of PTB and NTM before definite mycobacterial culture because of its great availability and short examination time [10]. The aim of this study is to compare and identify the chest CT characteristics that help to distinguish NTM lung disease from PTB in patients with AFB smear-positive sputum.

## Methods

### Patients

From January 2009 to April 2012, we received 9,267 sputum specimens from 3,958 patients at the laboratory of National Yang-Ming University Hospital (a 512-bed regional teaching hospital in Yilan, Taiwan) and the Far Eastern Memorial Hospital (a 1050-bed tertiary medical center in Taipei, Taiwan). Of these sputum specimens, 467 from 236 patients were AFB stain-positive. Only 134 of the AFB stain-positive patients had proven pulmonary TB, and 25 patients proven NTM lung diseases. A total of 95 CT scans obtained from the 159 patients were analyzed, 75 scans were from patients with PTB and 20 scans from NTM lung disease. The study was approved by the Institutional Review Boards of the National Yang-Ming University Hospital and the Far Eastern Memorial Hospital. All participants gave written informed consent and agree of information collection in the study. All images of the participants were taken as part of standard patient care.

### Diagnosis of pulmonary TB and NTM

All of the sputum specimens were treated with Ziehl-Neelsen staining. TB polymerase chain reaction (PCR) was performed with in-house IS6110-based PCR assays [11]. Mycobacterial cultures were performed using Löwenstein-Jensen medium [12]. Patients were diagnosed with pulmonary TB if M. tuberculosis was isolated from their sputum or tissue specimens. NTM was diagnosed according to mycobacterial cultures and the American Thoracic Society (ATS) guideline [13].

### CT scanning methods

The CT scans were original films obtained from each institution. All CT examinations were performed within 3 months of AFB smear test. Conventional CT scans were performed with a 5 mm slice-thickness at 5 mm intervals by using multi-detector CT (Philips Brilliance 64). HRCT images were obtained with a 1 mm slice-thickness at 10 mm intervals. Scanning extended from the lung apices to below the costophrenic angles at maximum inspiration. The CT scans were reviewed independently by two experienced radiologists and pulmonary specialists blinded to the patient’s microbiology results. The final decisions regarding the findings were determined by consensus.

### Statistical analysis

All statistical analyses were conducted using SPSS software for Windows (v 20; IBM Corporation, Armonk, NY, USA). Data are presented as frequencies for categorical variables, and by mean ± standard deviation for numerical variables. Categorical variables were compared using a chi-square test or Fisher’s exact test, and continuous variables were compared using an independent unpaired *t*-test. Univariate analysis was performed to evaluate the characteristic CT findings. Multivariate analysis was conducted using a logistic regression model to determine the independent predictive factors for patients with PTB and NTM infections. *P*-values of less than 0.05 were considered to be significant.

## Results

**Figure 1:**
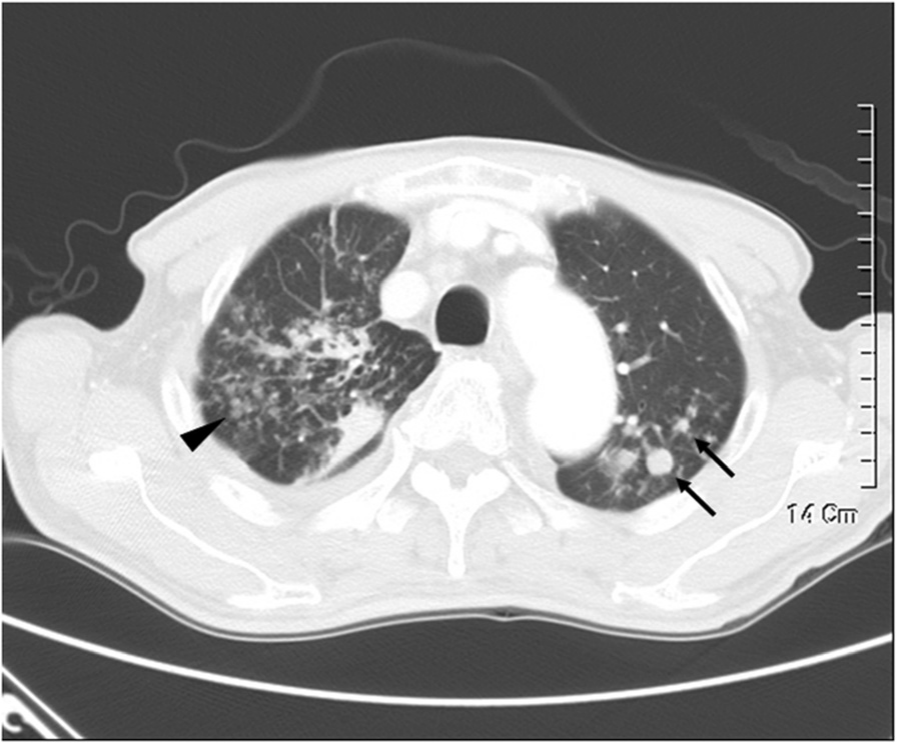
Thin section CT of a 72–year-old male with pulmonary tuberculosis demonstrating nodules (arrows) and tree-in-bud pattern (arrowhead); loculated pleural effusion at right lower lung zones.

**Figure 2:**
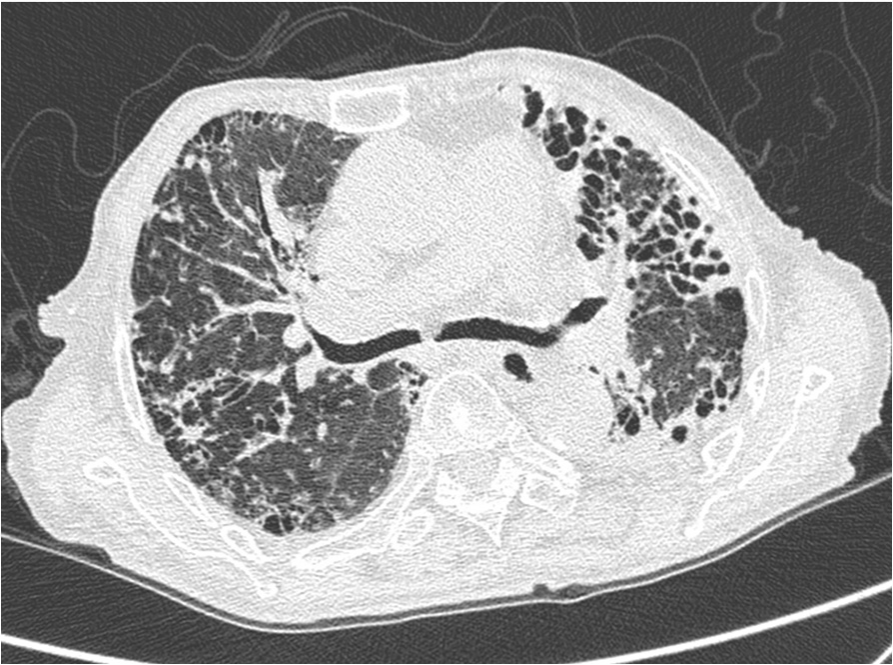
**High resolution CT with 1 mm slice-thickness of a 92 –year-old female with NTM (*Mycobacterium avium complex*) lung disease demonstrating the bronchiectasis and cystic changes at left upper and lower lobes and at right lower lobe.**

**Figure 3:**
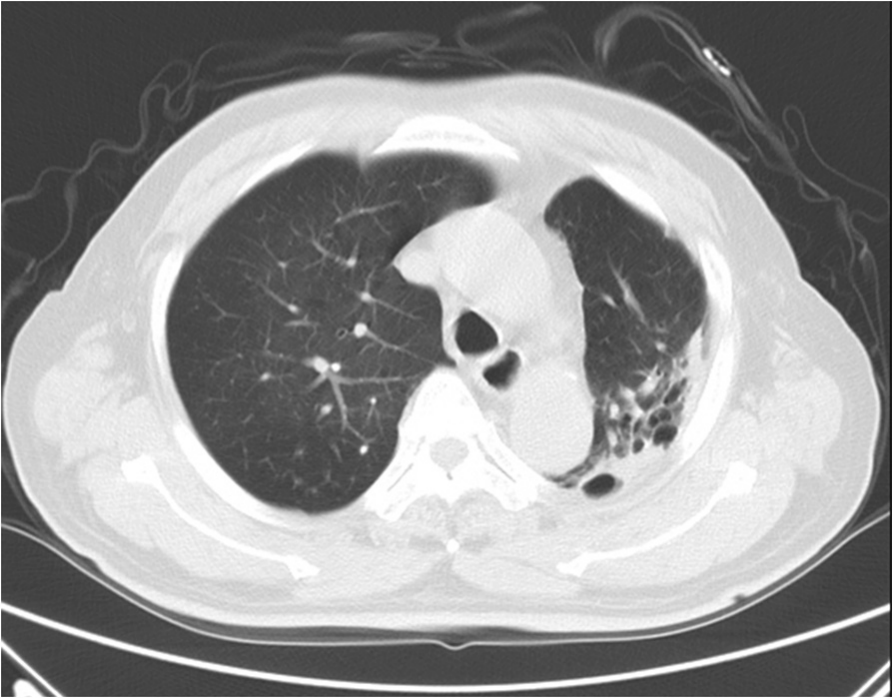
**Thin section CT of an 86 –year-old male with NTM (*Mycobacterium avium complex*) lung disease demonstrating the bronchiectasis and cystic changes at left upper lobe.**

The 75 patients with PTB included 58 men and 17 women with a mean age ± standard deviation of 65.6 years ±18.2 (range, 23–86 years). The 20 patients with NTM included 11 men and 9 women with a mean age ± standard deviation of 67.6 years ±14.6 (range, 30–92 years). The clinical data are summarized in Table 1. The CT findings were classified as presence or absence of pleural effusion, cavity formation, atelactasis, consolidation, nodules less than 10 mm in size, tree-in-bud pattern (branching centrilobular nodules), interlobular septal thickening, lung volume reduction, bronchiectasis, lymphadenopathy, calcified lymph nodes, as well as bronchiectasis and cystic changes. Of the 75 patients with PTB, 29 (38.7%) had pleural effusion vs. 3 (15.0%) in patients with NTM (*P* =0.047). 57 (76.0%) patients with PTB had nodules vs. 5 (25.0%) patients with NTM (*P* < 0.001). 61 (81.3%) patients with PTB had tree-in-bud opacities vs. 11 (55.0%) patients with NTM (*P* =0.021) (Figure 1). And 23 (31.1%) patients with PTB had cavities vs. 1 (5.0%) patient with NTM (*P* =0.018). Consalidation, lymphadenopathy and calcified lymph nodes were also more common in PTB patients. Of the 20 patients with NTM lung diseases, 4 (20.0%) had bronchiectasis and cystic changes vs. 3 (4.0%) patients with PTB; (*P* = 0.034) (Figures 2 and 3). Atelactasis, bronchiectasis without cystic changes and lung parenchymal volume reduction were more common in NTM lung diseases, but the univariate statistical results were not significant (Table 2). In multivariate analysis, CT scan findings of nodules was independently associated with patients with diagnoses of PTB (odds ratio [OR], 0.07; 95% confidence interval [CI], 0.02-0.30). Presence of bronchiectasis and cystic changes in CT scans was strongly associated with patients with NTM lung diseases (OR, 33.04; 95% CI, 3.01-362.55) (Table 3).

**Table 1:** Clinical characteristics of patients with pulmonary tuberculosis and NTM lung diseases

	**Pulmonary tuberculosis n = 75**	**NTM lung disease n = 20**	***P *value**
Age, years	65.57 ± 18.20	67.60 ± 14.63	0.647
Male	58 (77.3)	11 (55.0)	0.047
Ever smoker	24 (32.0)	3 (15.0)	0.134
Diabetes mellitus	14 (18.7)	3 (15.0)	1.000
Malignancy	3 (4.0)	0 (0.0)	1.000
Autoimmune disease	2 (2.7)	1 (5.0)	0.512
COPD	9 (12.0)	4 (20.0)	0.463
Pneumoconiosis	1 (1.3)	1 (5.0)	0.378

Numbers in parentheses are percentages. *NTM*: non-tuberculosis mycobacterium; *COPD*: chronic obstructive pulmonary disease.

**Table 2:** Comparative chest CT findings of non-tuberculous mycobacterial lung diseases and pulmonary tuberculosis

	**Pulmonary tuberculosis n = 75**	**NTM lung diseases n = 20**	***P *value**
Chest CT findings			
Pleural effusion	29 (38.7)	3 (15.0)	0.047
Cavities	23 (31.1)	1 (5.0)	0.018
Atelectasis	41 (54.7)	12 (60.0)	0.670
Consolidation	41 (54.7)	9 (45.0)	0.442
Random nodules	57 (76.0)	5 (25.0)	<0.001
Interlobular septal thickening	67 (89.3)	17 (85.0)	0.694
Bronchiectasis	10 (13.3)	4 (20.0)	0.484
Lung volume reduction	7 (9.3)	4 (20.0)	0.236
Tree-in-bud pattern	61 (81.3)	11 (55.0)	0.021
Lymphadenopathy	18 (24.0)	1 (5.0)	0.066
Calcified lymph nodes	5 (6.7)	0 (0.0)	0.580
Bronchiectasis and cystic changes	3 (4.0)	4 (20.0)	0.034

Numbers in parentheses are percentages. *NTM* = non-tuberculous mycobacterium.

*CT* = computed tomography.

**Table 3:** Multivariate analysis of predictors for NTM lung diseases

**CT findings**	**Odds ratio**	**95% CI**		
Bronchiectasis and cystic changes	33.035	3.010-362.552	Favor NTM	
Pleural effusion	0.198	0.038-1.031	Favor PTB	
Random nodules	0.074	0.019-0.296	Favor PTB	

*CI* = confidence interval; *PTB* = pulmonary tuberculosis; *NTM* = nontuberculous mycobacterium.

## Discussion

The present study shows that chest CT findings could help to differentiate pulmonary TB from NTM lung diseases. Upon comparing CT features between PTB and NTM, pleural effusion, nodules are significantly more common in patients with PTB and bronchiectasis combined with cystic changes are significantly more common in patients with NTM infections. In multivariate analysis, presence of nodules is significantly associated with PTB, and bronchiectasis combined with cystic changes are strongly associated with NTM lung diseases.

The definite diagnosis of PTB still depends on PCR or mycobacterial culture from bronchial washing or sputum. Clinical symptoms and image findings also play an important role in the diagnosis of TB. Typical radiological features of pulmonary TB include parenchymal diseases such as consolidation and cavities; pleural effusion, variable-sized nodules and lymphadenopathy are well documented and repeatedly reviewed in literatures. The CT findings in our study are mostly identical to the previously reported radiological findings of PTB. Familiar thin-section chest CT findings of nontuberculous mycobacterial infection include bronchiectasis, small and large nodules, centrilobular nodules, consolidation and fibrocavitary lesions [14,15]. Our study also demonstrates the common radiographic findings of bronchiectasis, tree-in-bud pattern, atelactasis and consolidation in patients with NTM lung diseases, but there are no significant differences as compared with the PTB patients. To our knowledge, the CT finding of bronchiectasis and cystic changes in NTM infection has not been emphasized in the previous studies. Although the radiological evidence of severe bronchiectasis and subsequent cystic changes, which is traditionally described as the honeycomb appearance, usually represents end-stage interstitial fibrosis, it is reported to be a dynamic process and may have different evolution [16,17]. In order to exclude the pre-existing bronchiectasis and cystic changes, the previous films of the four patients with NTM lung diseases demonstrating bronchiectasis and cystic changes were reviewed since the PACS implementation in our institutions from March 2004. Regarding the diagnosis of NTM, the isolation of NTM species from a respiratory sample is an insufficient evidence for the definite diagnosis of NTM lung disease. Some patients with positive NTM culture do not have evidences of pulmonary disease, and such infection may indicate colonization or transient infection. Therefore, the ATS issued diagnostic criteria for NTM lung diseases which not only rely on microbiological examinations, the clinical and radiographic evidences are also essential. Clinical researches have shown that disease attributable to NTM has a rising trend in many developed and developing countries [18,19]. In endemic areas, immediate and empirical treatment of mycobacterial diseases based on the result of AFB sputum smear has an important impact on disease transmission control. However, the administration of unnecessary empiric anti-TB treatment is not uncommon in real world practice and patients might suffer from the adverse effects of anti-TB drugs. Our colleagues (Shih-Chieh Chang, et al.) issued an article that revealed 40.4% of patients with positive sputum AFB stains did not have pulmonary TB in Taiwan [20]. According to Chang’s results, among patients who had positive sputum AFB stains without pulmonary TB, 25.8% were prescribed anti-TB treatment and 44% of them developed various adverse effects (hepatitis: 32%; skin rash: 20%). Although the adverse effects were usually not lethal, however, they did cause unnecessary harm. Our study provides radiographic evidence that chest CT finding of the bronchiectasis and cystic changes is statistically a strong indicator of NTM disease in patients with AFB smear-positive sputum. This characteristic CT feature is helpful to distinguish NTM from PTB in patients with equivocal clinical symptoms and pending culture results. Considering our results, physicians can make more precise decisions to initiate empiric anti-TB treatment and avoid unnecessary adverse effects on patients with final diagnoses of NTM lung diseases.

Our study had several limitations that are worth noting. Not all the AFB smear-positive patients with NTM lung diseases underwent chest CT. It was likely that the study was biased toward patients with severe infection who warranted further CT scans evaluation. In all cases, CT scans were obtained within 3 months of AFB smear tests. Some AFB smear-positive patients had received empirical anti-TB drugs. The CT findings in patients with actual PTB diagnoses might show improvement and somewhat not identical to the typical features of pulmonary TB. Finally, although there was a strong association of random nodules in PTB as well as bronchiectasis and cystic changes in NTM-LD statistically, the conclusions drawn by our statistical results needed a large-scale analysis to be confirmed.

## Conclusions

Our results suggest that pulmonary tuberculosis and NTM lung diseases can usually be distinguished by assessing the CT findings of nodules in patients with PTB as well as bronchiectasis and cystic changes with NTM. The CT distinction between these two mycobacterial infections may help radiologists and physicians to know the most likely diagnoses in AFB-smear positive patients and avoid unnecessary adverse effects and costs of anti-TB drugs in endemic areas.

## Competing interests

The authors declare that they have no competing interests.

## Authors’ contribution

MKY and CYC contributed equally to this work. MKY: study conception, design, and collection of data. CYC: drafting, submit and revise the manuscript. SCC: analysis and interpretation of data. PHT: study conception, design. Y-ML: acquisition and collection of data. JWH: acquisition and collection of data. All authors read and approved the final manuscript. All of the authors had access to the data and played a role in the writing this manuscript.

## Pre-publication history

The pre-publication history for this paper can be accessed here:

http://www.biomedcentral.com/1471-2466/14/65/prepub
